# Web-Based Intervention Using Self-Compassionate Writing to Induce Positive Mood in Family Caregivers of Older Adults: Quantitative Study

**DOI:** 10.2196/52883

**Published:** 2024-11-21

**Authors:** Farah Wiita, Aileen K Ho, Netta Weinstein

**Affiliations:** 1 School of Psychology and Clinical Language Sciences University of Reading Berkshire United Kingdom

**Keywords:** self-compassion, caregivers, mindfulness, intervention, writing, experimental

## Abstract

**Background:**

Caregiver burden can impact the mental health of family caregivers, but self-compassion may help reduce this impact. Brief self-compassion interventions have been shown to be useful but have not been tested in family caregivers of older adults.

**Objective:**

This study aimed to test the effects of a brief self-compassion intervention and its components (self-kindness, common humanity, and mindfulness) on mental well-being and mood when reflecting on difficult family caregiving experiences.

**Methods:**

British caregivers were recruited through a web-based panel. Three experimental studies manipulated the self-compassion intervention. In study 1 (n=206) and study 2 (n=224), participants wrote about a difficult caregiving experience while focusing on 1 self-compassion component (self-kindness, common humanity, or mindfulness). In study 3 (n=222) participants focused on all components. Self-compassion, serenity, guilt, and sadness were measured.

**Results:**

In studies 1 and 2, condition effects showed mindfulness unexpectedly lowered mood. Inconsistent and modest benefits to affect were achieved by engagement in self-kindness and common humanity in study 1 (guilt [lowered]: *P*=.02 and sadness [lowered]: *P*=.04; serenity [nonsignificantly raised]: *P*=.20) and also in study 2 (sadness [nonsignificantly lowered]: *P*=.23 and guilt [nonsignificantly lowered]: *P*=.26; serenity [raised]: *P*=.33); significant benefits for self-compassion and mood were found in study 3 (serenity [raised]: *P*=.01, kindness [raised]: *P*=.003, and common humanity [raised]: *P*≤.001; guilt [lowered]: *P*<.001 and sadness [lowered]: *P*≤.001). More intensive efforts should be made to promote self-compassion in caregivers of older adults, with caution advised when relying primarily on mindfulness approaches.

**Conclusions:**

Self-compassionate writing may be beneficial for family caregivers, but more intensive interventions are needed. Further research is needed to determine the optimal dosage and content for achieving the greatest effects.

## Introduction

### Background

Individuals may experience optimum health in older age, but for those experiencing age-related health challenges, the need to receive practical and sometimes extensive support from others can increase [1]; the responsibility often falls on family members [2]. This study explores an intervention to support positive mood for those family members who experience challenges, including psychological distress, which may result from the stresses of caregiving [3,4].

Caregiver “role strain” is defined within a stress process model as the experience of managing multiple demands, which can lead to the individual becoming overloaded with commitments [5-7]. This is because the provision of informal care places demands on others who may be in paid employment in addition to this role [8]. Furthermore, informal caregivers of older adults are often older adults caring for a parent, partner, or spouse [8] who are also at risk of age-related difficulties and could face their own physical and psychological challenges [9,10]. Informal caregiving also carries the risk of financial burden where work hours may be reduced to meet caring demands [8]. Caregivers may refuse opportunities, and work performance may be impacted by the demands of managing multiple roles.

Recognizing the needs of people who care informally for older adults, interventions are needed to improve their well-being [11]. Studies have shown that self-compassion interventions can improve the well-being of individuals in terms of outcomes such as depression and rumination [11-13]. However, to date, little research has focused on the needs of caregivers of older adults [11,13]. The focus of this study was to evaluate brief self-compassion writing interventions in carers of older adults aged ≥65 years, integrating these 2 lines of research.

### Self-Compassion

Self-compassion has been defined in multiple ways in the empirical literature. Early empirical work was rooted in Buddhism, where compassion was broadly defined as sensitivity to discomfort in self and others [14,15]. In Western psychology, the definition of the concept has been investigated, notably with compassion for self, for others, and from others being studied by Gilbert et al [14] and self-compassion being studied as a separate entity by Neff [15]. This paper will focus on self-compassion as defined by Neff [15]; this definition understands self-compassion to be composed of 3 components: self-kindness, common humanity, and mindfulness [15,16]. Although other approaches to self-compassion have been used to measure self-compassion (eg, [14]), the Self-Compassion Scale has been commonly used in research [12,15] and found to be particularly informative for work with caregivers in our earlier research [17].

The first component of self-compassion (self-kindness) involves responding to oneself with gentleness and understanding, allowing oneself to confront difficulties, inadequacies, and failings with acceptance and kindness [15]. The second component (common humanity) involves reflecting on difficulties as part of a shared experience, recognizing that all humans experience discomfort, are vulnerable, and have imperfections and that these difficulties are also endured by others [15].

Mindfulness is an essential component of self-compassion that supports the development of self-kindness and a sense of common humanity [15,18,19], which involves open awareness and acceptance of difficult thoughts and feelings [15]. With this awareness, individuals identify the source of their discomfort and approach their feelings in a gentle and soothing way (termed self-kindness). In addition, they better recognize and link their experience to those of others and identify experiences as part of the shared human experience (termed common humanity).

### Self-Compassion Interventions for Carers

Qualitative interviews with caregivers and professionals in aging and dementia care highlighted a tendency for caregivers to focus primarily on the needs of the care recipient [20]. This work also showed that a lack of self-compassion can lead to caregivers feeling overwhelmed, guilty, and ashamed. Furthermore, a cross-sectional study focused on informal caregivers of older adults demonstrated self-compassion and dispositional mindfulness to act as buffers for psychological distress [21]; in recent qualitative research, participants emphasized that caregivers should extend the same compassion they offer to others to themselves [20].

Outside the context of caregiving, previous intervention research focused on self-compassion has found promising improvements in outcomes including rumination and depression for those who receive self-compassion interventions [12]. However, there has been little research into the application of these or other interventions targeting self-compassion for informal caregivers of older adults [11,13], despite the need of this population for such an intervention. An integrative review of the literature identified only 4 studies focused on interventions for self-compassion in informal carers of older adults, none of which were statistically evaluated [13]. Of the 4 studies identified, 1 was a descriptive cross-sectional survey, 1 was qualitative, and the remaining 2 were randomized control trials measuring self-compassion as an outcome, with no preceding education or intervention for self-compassion specifically. Despite the lack of focus in this area, the authors noted that self-compassion could reduce caregiver burden because it may promote emotion regulation to help manage stress [11].

### Writing Interventions for Self-Compassion

Outside the context of caregiving, writing interventions have been useful for investigating the induction of mind state, including self-compassion. These interventions have been conducted over extended periods with the use of diary keeping and over shorter periods in the form of focused writing tasks, for example. From these interventions, improvements have been noted when targeting the following areas: (1) general self-compassion wherein participants write to themselves in a caring, supportive tone [22]; (2) writing with self-compassion based on the components by Neff [15] while focusing on a past emotionally challenging experience with common humanity, self-kindness, and mindfulness [23]; and (3) self-compassion focused on writing with only 1 component (ie, mindfulness, self-kindness, or common humanity) to investigate spillover effects across measured outcomes measured by each subscale [15,18]. The aforementioned studies produced some improvements in self-compassion, suggesting a writing intervention may provide an effective and flexible approach to investigating self-compassion components and how they may relate to a caregiver population.

### Recent Studies

Three studies explored the components of self-compassion as described by Neff [15]. Continuing the work of Dreisoerner et al [18], who explored spillover between the self-compassion components in an 8-week writing intervention, and Neff et al [24], who adapted and tested a state version of the self-compassion scales, this study focused on self-compassion as a state in caregivers.

Study 1 drew on recommendations to include mindfulness at the start of self-compassion interventions [15,18,19]. Self-compassion components were tested separately (self-kindness, common humanity, and mindfulness), with a mindfulness induction at the start of the procedure, to investigate differing effects on mood. In study 2, we looked at the self-compassion components separately, without a mindfulness induction, to understand their individual effects on mood. Finally, study 3 helped us to refine conclusions from studies 1 and 2, while testing an adapted induction method proposed by Neff et al [24].

All studies involved informal caregivers of older adults, with the first 2 studies including postintervention measures for mood and self-compassion, as for the research of Breines and Chen [25] and Miyagawa et al [26]. Study 3 included pre- and postintervention measures to align with the protocol of Neff et al [24].

The overall aim was to test the self-compassion components in a novel online intervention for caregivers of older adults. This study was then exploratory, although we did predict that engagement in each self-compassion component would benefit mood.

Another aim was to develop a widely accessible intervention with a web-based delivery. Recent findings have demonstrated promising emotional well-being outcomes for a digitally delivered mindfulness and self-compassion intervention with caregivers for patients with dementia [27]. Furthermore, digitally accessible interventions have the potential to disseminate self-compassion tools more widely compared with face-to-face delivery [28]. A study showed that technology provided vital support to mental well-being for older adults who were otherwise socially isolated [29]; social isolation is also a common experience for family caregivers [30]. This study contributes toward the development of a highly accessible, much-needed intervention for caregivers of older adults. In addition, research has found long-term benefits for short online interventions [31], which warrants testing this intervention for caregivers.

### This Study

To promote self-compassion, participants engaged in a brief writing exercise and then completed state measures assessing mood and self-compassion.

Because mindfulness has been identified as a precondition of self-compassion [15,18,19], we initially included mindfulness in our inductions of self-compassion and compared self-kindness and common humanity exercises with mindfulness writing alone and a neutral control group who wrote about the facts of their caregiving experience.

On the basis of the review of the literature, we hypothesized the 6 effects of brief writing interventions for our family caregiver samples.

Our first set of hypotheses concerned the efficacy of brief writing on self-compassion:

- Hypothesis 1: Writing with mindfulness would increase self-compassion.
- Hypothesis 2: Writing with self-kindness would increase self-compassion.
- Hypothesis 3: Writing with common humanity would increase self-compassion.

Our last set of hypotheses concerned the impact of brief self-compassionate writing on mood:

- Hypothesis 4: Writing with mindfulness would improve mood.
- Hypothesis 5: Writing with self-kindness would improve mood.
- Hypothesis 6: Writing with common humanity would improve mood.

## Study 1

### Methods

#### Ethical Considerations

The included studies were reviewed by the university research ethics committee and were granted favorable ethics approval (2021-193-AH). All included participants provided informed consent and were debriefed after the study. Participants’ data remained anonymous from the point of data collection and beyond because we recruited through Prolific [32], which uses a number identification system without providing names. Participants received payment for their time at the standard hourly Prolific rate, which was set at £7.50 (US $9.72) at the time of the study.

#### Participants

The sample size was calculated using G*Power (Universität Düsseldorf) [33]. Because the research was relatively novel, we did not have a reliable source to estimate effect size. Instead, we designed power to detect a moderate effect size of *f*=0.25. For a power of 0.90 at Cronbach α≤0.05, a sample size of 232 was needed to detect differences between the 4 conditions. Sensitivity analyses indicated the final number recruited following exclusions (n*=*206) reduced power to 0.86.

In total, 243 participants completed the study. Exclusions were applied where participants spent <8 minutes or had not completed a writing condition task; a previous online writing intervention lasting approximately 8 minutes was found to elicit improved outcomes [34]. Second, responses from participants who repeated the activity were removed, leaving 230 participants. Finally, responses where the completion time lasted >30 minutes were excluded, leaving 206 participants. We assumed longer completion times indicated participants had likely left their computers midtask making it difficult to measure state as intended. The remaining responses were included in the analyses.

For demographics, the mean participant age was 42.80 (SD 13.52; range 19-73) years. In the included sample (n=206), gender was reported as 47.6% (n=98) men, 50.5% (n=104) women, and 1.9% (n=4) nonbinary. Ethnicity was reported as 90.8% (n=187) White, 3.9% Asian (n=8), 1.5% (n=3) African or Caribbean, 1.9% (n=4) mixed, and 1.9% (n=4) other. Of the 206 participants, 24 (12%) cared for someone aged <65 (mean 45.88, SD 15.23; range 8-63) years. There were 88% participants (181/206) caring for recipients aged ≥65 (mean 80.03, SD 7.92; range 65-98) years. The mean number of years for caring was 5.30 (SD 5.16; range 1-37) years. The percentage of participants caring for a parent was 59.7% (n=123), for a sibling was 1% (n=2), for a spouse or partner was 4.4% (n=9), for a friend was 6.3% (n=13), and for others was 28.2% (n=58). The nature of care provided was mostly reported to involve supporting multiple needs (n=97, 47.1%), both physical and psychological. Diagnoses of multiple conditions (physical and psychological) were reported most often (n=64, 31.1%). In terms of living arrangements, 31.6% (n=65) of the participants reported living with the recipient, and 50.5% (n=104) of the participants received some professional caregiving support. Most participants (154/206, 74.8%) also engaged in paid work outside of caring.

Although this study aimed to focus on caregivers of older adults aged ≥65 years, some cared for other age groups. We included these participants because self-compassion in caregivers was likely to benefit informal caregivers across recipient groups who also faced high demands [35].

#### Procedure

Participants were recruited through Prolific [32] during February and March 2022 and the research was registered retrospectively at ClinicalTrials.gov (NCT06507826). Information sheets, instructions, consent forms, surveys, and writing exercises were available to participants through Qualtrics (version XM; Qualtrics) [36]. The initial survey asked for participants’ consent, demographic information, and details relevant to their caregiving situation. Participants received payment for their time through Prolific [32].

Participants were randomly assigned to 1 of the 4 conditions through Qualtrics [36] (control: 56/206, 27.18%; mindfulness: 54/206, 26.21%; mindfulness and self-kindness: 51/206, 24.76%; and mindfulness and common humanity: 45/206, 21.84%), delivered through writing exercises lasting a total of 8 minutes. This period was set according to a previous experimental study, which included sessions of 7 to 10 minutes of self-compassionate writing [18]. Writing exercises drew upon methods of previous research [15,16,18,22,23,37-39]. Two active writing conditions, 1 control, and 1 control with mindfulness, were adapted from writing exercises constructed by Dreisoerner et al [18], who drew on the work of Germer and Neff [39].

Instructions used by Dreisoerner et al [18] were adapted for writing focused on individual self-compassion components plus mindfulness, with instructions to recall a distressing care–related experience. All writing activities started with 4 questions targeted at the recollection of a difficult caregiving event, which occurred over the previous week. This approach was designed to elicit emotion and ground the discussion in a meaningful, self-relevant experience [23,37]. All participants completed this part. They were given 2 minutes per question within the control condition and 30 seconds per question within the remaining 3 conditions.

The control condition involved focusing on the difficult caregiving event itself. Participants were asked questions to elicit descriptive responses, for example, “What was happening in the situation?” and “What made the situation distressing?” In the mindfulness condition, participants first wrote about the difficult caregiving event and then spent 3 minutes engaging in mindful writing. Instructions in the mindfulness condition directed participants to write about the recalled difficult event, describing emotions they experienced without engaging in them. The examples of prompts to elicit responses for mindfulness included the following: “Spend time writing about how you felt in this situation” and “Do this whilst accepting these feelings without downplaying or dramatising the experience.” This approach encouraged participants to identify difficult feelings with understanding and acceptance rather than repression, allowing greater clarity of their experience [15]. This exercise was then designed to encourage participants to become aware of the situation from an unattached viewpoint, noticing all aspects of the situation without judgment or feeling overwhelmed [15].

Mindfulness exercises were also used together with the 2 self-compassion writing exercises (self-kindness and common humanity). In these 2 conditions, participants wrote about an event (as in the control condition), described mindfulness (as in the mindfulness condition), and then engaged in the self-compassion exercises appropriate for their condition assignment. These self-compassion conditions included three 1-minute focused writing exercises. For the self-kindness condition, participants were asked to write understanding and supportive comments to themselves, including positive and empowering words for their efforts. Questions to elicit responses for self-kindness included the following: “Focusing on the difficult situation you have identified; celebrate the efforts you have made in supporting the person you care for. Engage in soothing and supportive words.” and “Think of the way you managed this situation, expressing kindness towards yourself.” Finally, the common humanity condition involved focusing on how other caregivers would have experienced the same difficulties. Examples of prompts to elicit responses for common humanity included the following: “Consider how other carers would have responded in a similar way in this situation.” and “Remind yourself that other carers would have found the situation stressful.”

In accordance with the Checklist for Reporting Results of Internet E-Surveys [40], the number of screens presented to participants in online surveys should be reported to understand participant experiences. All participants were initially presented with 4 screens including information, consent, and demographic questions. For controls, 6 screens included information and spaces to complete the main task; for mindfulness, information and task completion space spanned 9 screens; self-kindness information and tasks spanned 11 screens; and common humanity spanned 11 screens. All participants completed mood and self-compassion measures on 1 screen following their tasks. All participants were given the opportunity to provide feedback after completing their tasks.

Surveys were piloted on a small subsample through Prolific [32] before opening the survey to the total sample.

#### Measures

##### Positive and Negative Affect Schedule for Serenity, Guilt, and Sadness

The full Positive and Negative Affect Schedule (PANAS) [41] for mood include 20 items in total. We selected the serenity, guilt, and sadness subscales based on their relevance to a caregiver population. The PANAS scales [41] were also used in the research of Neff et al [24] as indicators of mood in relation to measured self-compassion.

Affect was measured with these 3 subscales as follows: serenity comprised 3 items on which participants rated their sense of feeling calm or peaceful for serenity, guilt included 5 items on which participants rated feelings of guilt or dissatisfaction with self, and sadness included 5 items related to unhappy feelings. A sixth item “dissatisfied with self” from the guilt subscale was omitted in error. Items were rated on a scale of 1 (very slightly or not at all) to 5 (extremely). High reliability was noted across all subscales (Cronbach α≥0.65). Cronbach α values for scale totals were as follows: serenity, Cronbach α=0.94; guilt, Cronbach α=0.92; and sadness, Cronbach α=0.91.

##### State Self-Compassion Scale Short Form

The 6-item State Self-Compassion Scale Short Form (SSCS-S) [24] was used to measure global state self-compassion. Participants rated the relevance of positive and negative statements related to self-compassion. An example of a positive statement was “I’m giving myself the caring and tenderness I need;” an example of a negative statement was “I’m obsessing and fixating on everything that’s wrong.” Participants rated items on a scale of 1 (not at all true for me) to 5 (very true for me). For the total SSCS-S, Cronbach α=0.79.

#### Data Analysis

Data were analyzed using SPSS (version 28; IBM Corp) [42]. Pearson correlation tests were used to explore associations between scale items. ANOVA tests were conducted to test condition effects on outcome measures.

### Results

#### Correlations

Multimedia Appendix 1 includes Pearson correlations, means, and SDs for composite scores on the discussed scales. All correlations were significant (*P*<.001). The SSCS-S was positively correlated with serenity (*r*_204_=0. 46). Negative correlations were found for the SSCS-S with guilt (*r*_204_=–0.37) and with sadness (*r*_204_=–0.49).

#### Analyses for Condition Effects

Tests of normality for scales for each condition showed that while some scores crossed +1 or –1 for skewness or kurtosis, scores did not cross this threshold for 20 out of 24 measured variables. Furthermore, of those that violated normality, the greatest was 1.58 for skewness and 1.78 for kurtosis. The threshold of +1 or –1 was recommended to determine normality distribution [43] and applied by Neff et al [24]. However, ANOVA tests are robust even with nonnormal distributions [44]. Because nonnormality was minimal and affected only a small proportion of the data, parametric ANOVA tests were used for all effect comparisons.

Multimedia Appendix 2 includes results for 1-way ANOVAs for condition effects for all scales. Significant effects were found between scores for guilt (*F*_3,202_=3.40; *P*=.02) and sadness (*F*_3,202_=2.78; *P*=.04). A Tukey honestly significant difference test was used to compare means for guilt. Results showed a significant difference in condition effects for self-kindness (mean 1.76, SD 0.88) and mindfulness (mean 2.33, SD 1.02; *P*=.01). Owing to unequal variance between groups for sadness scores (Levene *F*_3, 202_=3.01; *P*=.03), a Games-Howell post hoc test was conducted. This also showed significant condition differences between self-kindness (mean 2.10, SD 0.95) and mindfulness (mean 2.68, SD 1.22; *P*=.04).

## Study 2

### Methods

#### Ethical Considerations

Amendments for the study 1 protocol were requested for study 2. These amendments were approved by the university research ethics committee. Consent, anonymity, and payment for participants followed the same procedure as for study 1.

#### Participants

Because the number of conditions remained the same, power was based on the calculation for study 1. A total of 238 informal caregiver participants were recruited through Prolific [32] in July 2022. One exclusion was applied because the participant was a professional caregiver and did not meet the eligibility criteria. Further exclusions were applied for incomplete responses (9/238, 3.78%) and responses taking >30 minutes (4/238, 1.68%), reducing the number to 224 and power to 0.89.

The mean age of the included participants was 43.21 years (SD 13.36; range 18-70) years. In the included sample (n=224), gender was reported as 46.9% (n=105) men, 52.7% (n=118) women, and <1% (n=1) other gender. For ethnicity, 81.3% (n=182) reported as White, 9.4% (n=21) Asian, 2.7% (n=6) African or Caribbean, 3.6% (n=8) mixed, and 3.1% (n=7) other. The mean age of care recipients for this sample was 78.27 (SD 8.26; range 65-90) years. The mean reported years of caring was 5.34 (SD 4.46; range 1-30) years (n=224). For the reported relationship with the care recipient, 60.3% (135/224) were caring for a parent, <1% (2/224) for a sibling, 3.6% (8/224) for a spouse or partner, 7.6% (17/224) for a friend, and 27.7% (62/224) for others. For the nature of care provided, participants mostly reported supporting daily living tasks (206/224, 92%) such as assistance with shopping, cooking, and cleaning. Conditions causing restricted mobility were the most frequently diagnosed in this sample (142/224, 63.4%). For living arrangements, 27.2% (61/224) reported living with the recipient and 55.8% (125/224) received additional support. Most participants (164/224, 73.2%) were engaged in work outside of caring.

#### Procedure

The same protocol was followed as for study 1, but it was amended, with an expected completion time of 10 minutes. This time, participants were informed that they would have a set time to complete the exercise. For simplicity, demographic questions regarding recipient diagnoses, nature of care, and occupation were presented as multiple choice, with options based on responses from study 1.

The 4 induction exercises were set at 30 seconds per question and 90 seconds per question for the 3 self-compassion exercises. Mindfulness was removed from the start of self-kindness (n=55) and common humanity (n=54) conditions for study 2. For the control condition (n=58), participants spent one and a half minutes on each of the 4 questions. Finally, for mindfulness (n=57), participants spent 30 seconds per question for 4 induction exercises, followed by 2 minutes of mindfulness, and then an additional 2 minutes focusing on the induction exercise.

All participants were initially presented with 8 screens including information, consent, and demographic questions. For controls, 6 screens included information and spaces to complete the main task; for mindfulness, information and task completion space spanned 9 screens; self-kindness information and tasks spanned 10 screens; and common humanity spanned 10 screens. All participants completed mood and self-compassion measures over 3 screens following their tasks. All participants were given the opportunity to provide feedback after completing their tasks.

Surveys were piloted on a small subsample through Prolific [32] before opening the survey to the total sample.

#### Measures

##### Overview

Following each condition, participants completed questions for 4 scales and subscales (as for study 1). The presentation of scale items was randomized within Qualtrics [36] to control for order effects. We did include a manipulation check to test whether participants’ responses conformed to the presented tasks. However, no meaningful responses were found with this check, so details are not discussed here. Findings for the scales are discussed subsequently.

##### PANAS Serenity, Guilt, and Sadness

Subscales for serenity, guilt, and sadness were identical to study 1. The sixth item “dissatisfied with self” from the guilt subscale was omitted in error. High reliability was noted across all subscales, with Cronbach α≥0.65. Cronbach α values for scale totals were as follows: serenity, Cronbach α=0.94; guilt, Cronbach α=0.93; and sadness, Cronbach α=0.92.

##### State Self-Compassion Scale Long Form

The 18-item State Self-Compassion Scale Long Form (SSCS-L) was used to measure the 6 components of state self-compassion. Subscales included self-kindness, self-judgment, common humanity, isolation, mindfulness, and overidentification. Self-judgment, isolation, and overidentification were reversed scored according to the instructions by Neff et al [24]. The combined subscales were then used to find overall state Self-Compassion Scale scores. Participants rated statements in the same way as for the SSCS-S, with high reliability for individual subscales (self-kindness: Cronbach α=0.78, self-judgment: Cronbach α=0.78, common humanity: Cronbach α=0.85, isolation: Cronbach α=0.77, mindfulness: Cronbach α=0.78, and overidentification: Cronbach α=0.81). For the overall SSCS-L score, Cronbach α=0.83.

#### Data Analysis

Data were analyzed using SPSS [42].

### Results

#### Correlations

Multimedia Appendix 3 shows Pearson correlations, means, and SDs for composite scores on the discussed scales. All were significant, except common humanity with serenity, guilt, sadness, judgment, and overidentification. There were no significant effects for serenity, except with sadness (*r*_222_=−0.15; *P*=.03) and mindfulness (*r*_222_=0.17; *P*=.01).

Due to high skew and kurtosis for serenity, nonparametric Spearman rank correlations were also conducted for this scale. Results for Spearman correlations with serenity are as follows: mindfulness, *r*_222_=0.64; overidentification, *r*_222_=0.29; self-kindness, *r*_222_=0.61; judgment, *r*_222_=0.38; common humanity, *r*_222_=0.29; isolation, *r*_222_=0.43; SSCS-L, *r*_222_=0.59; guilt, *r*_222_=–0.43; and sadness, *r*_222_=–0.49. All Spearman correlations were significant (all *P* values ≤.001).

#### Analyses for Condition Effects

No significant condition differences were found across all scales (Multimedia Appendix 4).

Serenity scores were notably lower for the mindfulness condition, although not significantly. For serenity, high levels of skew and kurtosis were noted specifically for the mindfulness condition (skewness=−7.471, SD 0.32 and kurtosis=56.227, SD 0.62). Because of this, a nonparametric test was also conducted for scores on this scale. Nonparametric Kruskal-Wallis results were nonsignificant, indicating no effects were found (*H*_3_=1.53; *P*=.68). Median scores were lowest for the mindfulness condition (median 2.67, IQR 1.66-3.83), followed by control (median 2.83, IQR 2.00-4.00), with no difference between self-kindness (median 3.00, IQR 2.00-4.00) and common humanity conditions (median 3.00, IQR 2.00-3.67).

Post hoc comparisons explored differences in serenity between the mindfulness and self-kindness conditions and the mindfulness and common humanity conditions. Due to high skew and kurtosis in the mindfulness condition for serenity, Mann-Whitney *U* tests were conducted. Differences were nonsignificant between mindfulness and self-kindness (*U*=1377; *P*=.27). Nonsignificant differences were also found between mindfulness and common humanity for serenity (*U*=1429; *P*=.34).

Further post hoc tests were conducted to compare both self-compassion (self-kindness and common humanity) and both control groups (control and mindfulness). A Mann-Whitney *U* test (for serenity scales due to skew and kurtosis) and independent 2-tailed *t* tests showed significant differences between groups for self-kindness (controls combined: mean 2.75, SD 0.99; self-compassion combined: mean 3.01, SD 0.96; *t*_222_=−2.05; *P*=.042) and self-judgment (controls combined: mean 3.08, SD 0.98; self-compassion combined: mean 3.35, SD 0.98; *t*_222_=−2.00; *P*=.05).

## Study 3

### Methods

#### Ethical Considerations

Amendments for the study 1 protocol were requested for study 3. These amendments were approved by the university research ethics committee. Consent, anonymity, and payment for participants followed the same procedure as for study 1.

#### Participants

Power was calculated for the inclusion of 3 groups using G*Power [33]. As for the previous studies, we calculated power to detect a moderate effect size of *f*=0.25. For a power of 0.90 at Cronbach α≤0.05, a sample size of 207 was needed to detect differences between the conditions. After exclusions, a final sample size of 222 participants was reached, yielding a statistical power of 0.92.

Data were collected through Prolific [32] in November 2022. The sample recruited included 325 informal caregiver participants. Of these participants, 306 responses were retained following exclusions according to participants’ ratings on a compliance measure [24]. A further 84 exclusions were applied where activities were incomplete or where <200 characters were included in each written response. This latter criterion was drawn from the procedure used by Neff et al [24]. One participant cared for someone just below the age for inclusion (aged 64 years instead of 65 years). This participant was retained.

Responses to our demographic survey indicated that the mean age for participants was 42.29 (SD 13.20; range 18-77) years. In the included sample (n=222), gender was reported as 49.5% (n=110) men, 50% (n=111) women, and <1% (n=1) nonbinary. Ethnicity included 82% (182/222) White, 7.7% (17/222) Asian, 3.2% (7/222) African or Caribbean, 4.1% (9/222) mixed, and 3.2% (7/222) other. The mean number of years for caring was 5.81 (SD 6.80; range 0-75) years. The mean age of care recipients for this sample was 78.94 (SD 8.34; range 64-90) years. Care was provided by 62.2% (138/222) of the sample for a parent, 2.3% (5/222) for a spouse or partner, 4.1% (9/222) for a friend, <1% (1/222) for a sibling, and other was reported by 30.6% (68/222) of the sample. The nature of care provided mostly involved help with daily living (208/222, 93.7%) such as cleaning and shopping. Diagnoses of conditions affecting mobility (139/222, 62.6%) were reported most often. In terms of living arrangements, 31.1% (69/222) of the participants reported living with the recipient, and 52.7% (117/222) of the participants received some professional caregiving support. Many participants (151/222, 68%) engaged in additional work besides caring.

#### Procedure

The same protocol was followed as for studies 1 and 2. Following procedures applied in the second study of Neff et al [24], compliance measures and demographic information were collected after writing exercises. Compliance measures were adapted from the studies by Neff et al [24] and Neff [45].

Participants were asked to recall a difficult caregiving experience, complete scales to measure self-compassion and mood, and then engage in their allocated written component. They were then randomly assigned to 1 of 3 conditions: a control condition (n=75), self-compassion condition (n=73), and a self-compassion without mindfulness condition (n=74). The control and self-compassion conditions were adapted from the recommended content of Neff et al [24] (eg, [46]).

Writing elements were structured as follows: (1) the control condition included 3 writing components to parallel the self-compassion condition but with descriptive content; (2) the self-compassion condition included writing with mindfulness, kindness, and then common humanity; and (3) the self-compassion without mindfulness condition included the same content as for self-compassion but with the removal of mindfulness. Following both self-compassion conditions, participants were asked to read through and reflect on their writing before completing the compliance measures, repeating the scales, and completing demographic information.

All participants were initially presented with 5 screens including information and consent. For controls, 4 screens included pretask measures, information, and spaces to complete the main task; the self-compassion pretask measures, information, and task completion space spanned 3 screens; and self-compassion without mindfulness pretask measures, information, and tasks spanned 3 screens. All participants completed posttask mood and self-compassion measures and demographic questions presented over 6 screens following their tasks. All participants were given the opportunity to provide feedback after completing their tasks.

Surveys were piloted on a small subsample through Prolific [32] before opening the survey to the total sample.

Although the timing for completion of the study was not restricted, the estimated completion time was 14 minutes. To ensure participants included the minimum amount of required writing (at least 200 characters per writing exercise), instructions to write at least 3 lines per question were given. After the first 10 participants, it was noted that their answers often did not meet the required length. One participant revealed that they had completed the study on a phone. Because a mobile phone screen was smaller and the display would differ, instructions to complete the study on a desktop were emphasized. To increase the salience of this requirement, a note was added asking participants to complete the survey on a desktop only. Instructions for the amount of writing were increased to 5 lines per question. As for the previous studies, an opportunity to provide feedback was included at the end.

#### Measures

##### Compliance

Compliance measures followed the recommendations of Neff et al [24] and Neff [45]. For both the self-compassion and self-compassion without mindfulness conditions, compliance was assumed where participants selected an option that indicated the task was approached with self-compassion (eg, [24,45]).

##### PANAS Serenity, Guilt, and Sadness

Serenity, guilt, and sadness subscales were used as in studies 1 and 2. The sixth item for guilt “dissatisfied with self,” which was erroneously omitted for studies 1 and 2, was included for study 3. High reliability was noted across all subscales and overall scores for times 1 and 2. For time 1, Cronbach α values were as follows: serenity, Cronbach α=0.93; guilt, Cronbach α=0.93; and sadness, Cronbach α=0.93. For time 2, Cronbach α values were as follows: serenity, Cronbach α=0.87; guilt, Cronbach α=0.94; and sadness, Cronbach α=0.92.

##### Scales for Self-Compassion

The 18-item scale was retained, with scoring conducted as for study 2. All Cronbach α subscale totals were ≥0.65. Cronbach α values for individual subscales were as follows: self-kindness, Cronbach α=0.66; self-judgment, Cronbach α=0.79; common humanity, Cronbach α=0.76; isolation, Cronbach α=0.81; mindfulness, Cronbach α=0.84; and overidentification, Cronbach α=0.75. For the entire SSCS-L scale at time 1, Cronbach α=0.86.

At time 2, Cronbach α subscale totals were ≥0.65. Cronbach α values for individual subscales were as follows: self-kindness, Cronbach α=0.89; self-judgment, Cronbach α=0.80; common humanity, Cronbach α=0.81; isolation, Cronbach α=0.88; mindfulness, Cronbach α=0.82; and overidentification, Cronbach α=0.76. The Cronbach α for the overall SSCS-L at time 2 was 0.84.

#### Data Analysis

Data were analyzed using SPSS [42]. Repeated ANOVA tests were used for the comparison of scores across times 1 and 2.

### Results

#### Correlations

Multimedia Appendix 5 displays Pearson correlations, means, and SDs for composite scores on the discussed scales. Most were significant at both time 1 and 2 (*P*<.001). A strong negative correlation was found between isolation and sadness at times 1 and 2, respectively (*r*_220_=−0.71; *P*<.001 and *r*_220_=−0.79; *P*<.001). Judgment and overidentification showed strong positive correlations for times 1 and 2, respectively (*r*_220_=0.63; *P*<.001 and *r*_220_=0.68; *P*<.001).

For within-subjects scores for participants at each particular time point, mean scores show that the negative correlation found for isolation and sadness was explained by decreased sadness at time 2 and increased isolation at time 2. For between-subjects scores for participants at each particular time point, sadness increased at time 2 for control but decreased for both self-compassion conditions, whereas isolation increased in all conditions at time 2.

#### Effects of Condition Across Time

Skew and kurtosis were mostly within or close to +1 or –1, with the greatest skew for guilt time 2 (*skewness*=1.84, SD 1.03) and greatest kurtosis for overidentification time 2 (*kurtosis*=−1.01, SD 0.92). Levene tests were not violated. Parametric tests were then used.

Results for condition effects across time (condition×time interactions) with means and SDs are summarized in Multimedia Appendix 6. Condition differentially changed across time when predicting serenity (*F*_2,218_=4.55; *P*=.01), guilt (*F*_2,219_=9.85; *P*≤.001), and sadness (*F*_2,219_=11.48; *P*≤.001). Significant results were also present, predicting kindness (*F*_2,219_=6.10; *P*=.003) and common humanity (*F*_2,219_=4.59; *P*=.01). There were no significant differences for condition changing across time for overall SSCS-L scores.

#### Follow-Up Effects Within Subjects

Within-subjects ANOVAs for times 1 and 2 showed significant effects for the self-compassion condition on all self-compassion components and the overall SSCS-L (Multimedia Appendix 7).

For self-compassion, improvements were found for mindfulness (*F*_1,72_=7.09; *P*=.01), overidentification (*F*_1,72_=11.62; *P*≤.001), self-kindness (*F*_1,72_=23.86; *P*=.001), self-judgment (*F*_1,72_=10.65; *P*=.002), common humanity (*F*_1,73_=19.80; *P*≤.001), isolation (*F*_1,72_=14.84; *P*≤.001), and total SSCS-L (*F*_1,72_=31.62; *P*≤.001). For mood, the self-compassion group improved on guilt (*F*_1,72_=12.56; *P*≤.001) and sadness (*F*_1,72_=11.78; *P*≤.001).

Significant improvements were found for the self-compassion without mindfulness condition on the same measures: mindfulness (*F*_1,73_=20.53; *P*≤.001), overidentification (*F*_1,73_=8.72; *P*=.004), self-kindness (*F*_1,73_=28.78; *P*≤.001), self-judgment (*F*_1,73_=4.25; *P*=.043), common humanity (*F*_1,73_=27.85; *P*≤.001), isolation (*F*_1,73_=13.52; *P*≤.001), and SSCS-L (*F*_1,73_=40.14; *P*≤.001). For mood, self-compassion without mindfulness improved for guilt (*F*_1,73_=37.45; *P*≤.001) and sadness (*F*_1,73_=30.02; *P*≤.001).

Controls also showed improvements, albeit more modest improvements, for mindfulness (*F*_1,74_=18.96; *P*≤.001), self-judgment (*F*_1,74_=14.51; *P*≤.001), common humanity (*F*_1,74_=4.87; *P*=.03), isolation (*F*_1,74_=4.34; *P*=.04), and total SSCS-L (*F*_1,74_=13.92; *P*≤.001). There were negative effects for mood with lowered serenity (*F*_1,74_=6.83; *P*=.01).

## Discussion

### Principal Findings

Three studies were conducted to investigate self-compassion in caregivers of older adults. Because little previous research had focused on this area, the first 2 studies focused on testing each of the separate components of self-compassion proposed by Neff [15]. We investigated the effects of each component of self-compassion to explore their independent contributions toward measured outcomes. The first 2 studies showed weak and inconsistent benefits of self-compassion over mindfulness alone. In study 3, some support for hypotheses 1 to 3 (greater self-kindness and common humanity) and hypotheses 4 to 6 (lower sadness and guilt and higher serenity) was noted, where more consistent benefits of engaging in writing about caregiving experiences were found when more complete self-compassion was incorporated within the writing. However, the benefits were not greater when examining the mindfulness component of self-compassion or the overall self-compassion scores.

In study 1, we included mindfulness at the start of self-compassion conditions to engage participants in their recalled events [18,24]. However, self-compassion was not significantly increased using this method. Instead, we found that guilt and sadness increased in the mindfulness condition compared with the self-compassion condition. However, it should be noted that, although participants were asked to time their responses, these timings were not controlled. It is possible then that variations in time spent on each task may have influenced outcomes.

The first goal for study 2 was to investigate the effects of self-compassion alone by removing mindfulness from the self-kindness and common humanity conditions to understand their effects on mood. Similar to findings from study 1, we found that serenity was lower in the mindfulness condition for study 2. Counter to expectations, our findings for study 2 suggested that mindfulness was not beneficial but potentially detrimental for caregivers included in our sample. However, we were unable to conclude detrimental effects with confidence because no significant differences were found with post hoc tests.

Study 2 procedures intentionally limited engagement time to standardize it across conditions. However, this methodological decision may have hindered participants’ self-expression and reduced potential benefits for those who were more deeply engaged in the activity.

It should be noted that, although mindfulness includes benefits such as clarity and acceptance of difficult emotions as described by Neff [15], engaging with these emotions could be challenging and potentially harmful for some individuals [47]. Returning to our results for study 1, increased guilt and sadness were likely due to the focus on difficult care experiences required in the mindfulness condition without engagement in self-kindness and common humanity. In study 2, the effects on serenity may have been due to the pressure of writing under timed conditions while also focusing on a difficult event, again with no engagement with self-kindness and common humanity.

The third goal of study 2 was to replace the SSCS-S with the SSCS-L [24] to examine whether this comprehensive measure would be more sensitive to condition effects. Indeed, from validation studies of the long version of the self-compassion scales (both state and trait), these measures were suggested to be useful for looking at effects within the individual self-compassion components [24,48]. However, post hoc *t* tests for both self-compassion conditions combined demonstrated that weak condition effects may have been present but not detectable across the 4 conditions.

Study 3 was designed primarily to adapt more closely to methods used by Neff et al [24], while addressing the limitations of study 2. First, we tested the intervention without a timer to allow participants to engage in activities and eliminate this potential distraction. We also reduced the number of times participants switched between writing activities to allow participants to engage more deeply in the tasks. A third goal was to revisit the writing instructions, taking note of those suggested by Neff et al [24]. Because the initial instructions by Neff et al [24] asked participants to complete all self-compassion components as 1 condition, we included an additional control condition without the mindfulness component to address the negative effects observed in studies 1 and 2. Finally, new to this study, we measured mood before and after the writing exercise to examine within-person change.

In study 3, the expectations that self-compassionate writing would increase self-compassion were supported in both self-compassion conditions, with the greatest effects for the self-compassion condition with mindfulness. However, improvements were noted for 2 self-compassion components separately (self-kindness and common humanity), with no greater improvements for mindfulness or total self-compassion scores. Findings showed that self-compassion increased regardless of the inclusion of mindfulness within the exercise.

A notable finding for study 3 was a significant increase for the control condition on 5 measures for self-compassion and total self-compassion measured on the SSCS-L. However, there was also a significant decrease in serenity scores for this condition, suggesting that writing alone may have reduced peaceful mood. This potential downside was not observed when self-kindness and common humanity were included in the intervention. Merely describing an event may negatively influence some elements of self-compassion and mood, but elements of self-compassion can help mitigate these effects.

Focusing on the potential harms of mindfulness, caution has been noted on the use of mindfulness-based intervention and practice (eg, [47,49-51]), although there has been little research in this area. Harms from interventions have been defined as a sustained detrimental outcome directly resulting from treatment [47,52]. However, because mindfulness appeared to enhance benefits for participants in study 3 in this research, this suggests that when applied together with the other self-compassion components, mindfulness could be beneficial.

Looking at differences across outcomes according to writing conditions in this study, previous research using interventions presented over an extended period may offer further insight. In line with our findings from study 3, the successful application of mindfulness-based interventions has been widely documented [47]. Furthermore, it was suggested that therapeutic exposure to a difficulty could have temporary effects, which later elicit greater long-term benefits [47,53]. For example, mindfulness was included in the mindfulness self-compassion program, which was presented over an 8-week period [38]. In this program, mindfulness was identified as a necessary component to achieve beneficial outcomes. An extended 8-week program was also the protocol applied by Dreisoerner et al [18] in their study in which participants completed activities for self-compassion. Furthermore, findings from a meta-analysis indicated that writing interventions spaced over time were most effective for achieving beneficial outcomes [54]. In this research, further studies should measure the effects of the intervention over time to understand longer-term outcomes and enhance the potential for lasting benefits..

Drawing on the previous application of interventions using self-compassion, studies have found improvements such as increased resilience, mood, health behaviors, and self-care [55-58]. In the interest of caregivers, self-compassion may then play a role in protecting the well-being of caregivers by providing valuable tools for managing and coping with the situation [9]. Specifically, our study found prominent improvements in the self-kindness and common humanity components of self-compassion. It is recognized in the literature that caregivers can experience a sense of self-judgment or guilt [4,59]. Self-kindness offers an alternative way to treat oneself when one identifies their decisions, thoughts, or behaviors as incorrect or potentially unhelpful for the care recipient [15]. Self-kindness may be particularly useful when faced with challenges where the caregiver experiences little or no control, such as observed deterioration in the care recipient [60]. In addition, family caregiving can involve less opportunity to observe others in similar situations due to the requirement to provide one-to-one care [61]; common humanity offers an alternative perspective through recognition that other carers can also face challenges [15].

On the basis of these studies, recommendations for research into the successful application of self-compassion interventions for family carers of older adults include considering potential health challenges and needs of caregivers as well as limitations to what they can realistically provide the recipient. For example, those caring for older adults informally have often been noted to be middle-aged or older adults providing care for an older spouse, partner, or parent [8]. Interventions may need to include tasks that also address the management of caregivers’ own age- or health-related challenges. Research in this area should also take account of the individual needs of the caregivers.

### Limitations and Future Directions

In this study, self-compassionate writing exercises were completed in a short exercise at 1 time point. It would be interesting to examine the effects on the mood of the caregivers after completing these tasks over a longer time scale. In addition, the study was conducted remotely but could also be presented in a controlled environment in a future work, for example, by inviting participants to a laboratory. Furthermore, it would be useful to measure the amount of time spent on each writing task to understand whether engagement intensity influences outcomes. Although in our studies, the time spent on writing sessions appeared unrelated to measured improvements, a meta-analysis [54] suggests that longer time spans may not necessarily lead to greater benefits.

### Conclusions and Recommendations

The 3 studies investigated the impact of a series of self-compassion interventions among caregivers of older adults. Across studies, mindfulness writing had mixed effects on self-compassion and mood but held the potential to benefit self-compassion writing (study 3). Conversely, writing from the perspective of both self-kindness and common humanity showed neutral to beneficial effects on self-compassion and mood, suggesting that these can be harnessed in more intensive interventions to improve caregiver well-being. We recommend that brief and remotely conducted self-compassionate writing interventions for older adult caregivers include self-kindness, common humanity, and mindfulness to achieve the best improvement profile and potential impact for better well-being.

## Appendix

Multimedia Appendix 1 Correlations for scale composites: study 1.

Multimedia Appendix 2 One-way ANOVA scores with means and SDs for all conditions: study 1.

Multimedia Appendix 3 Correlations for scale composites: study 2.

Multimedia Appendix 4 One-way ANOVA scores with means and SDs for all conditions: study 2.

Multimedia Appendix 5 Correlations for scale composites: study 3.

Multimedia Appendix 6 Repeated measures ANOVA scores for condition×time effects with means and SDs by condition: study 3.

Multimedia Appendix 7 Simple slope change for study 3.

## Abbreviations

SSCS-L: State Self-Compassion Scale Long Form
SSCS-S: State Self-Compassion Scale Short Form

## Data Availability

The datasets generated during this study are available in the Open Science Framework repository [62].
