# Supplementary material for: Web-Based Intervention Using Self-Compassionate Writing to Induce Positive Mood in Family Caregivers of Older Adults: Quantitative Study
Source: JMIR Form Res. 2024 Nov 21;8:e52883. doi: 10.2196/52883 (PMC11621718; doi:10.2196/52883)
Supplement: Multimedia Appendix 1 [file formative_v8i1e52883_app1.pdf]

Online Intervention using Self-Compassionate Writing to Induce Positive Mood in Family Caregivers of Older Adults

Appendix 1

Correlations for Scale Composites: Study 1 (*N* = 206)

	M (SD)	1	2	3
1. Serenity	2.73 (1.05)	-	-	-
2. Guilt	2.01 (0.96)	-.41[a]	-	-
3. Sadness	2.45 (1.07)	-.56 [a]	.54 [a]	-
4. SSCS-S	3.10 (0.81)	.46 [a]	-.37 [a]	-.49 [a]

Notes: SSCS-S – Self-Compassion Scale – Short Form.

[a] Statistically significant $P < .001$.
