# Supplementary material for: Web-Based Intervention Using Self-Compassionate Writing to Induce Positive Mood in Family Caregivers of Older Adults: Quantitative Study
Source: JMIR Form Res. 2024 Nov 21;8:e52883. doi: 10.2196/52883 (PMC11621718; doi:10.2196/52883)
Supplement: Multimedia Appendix 2 [file formative_v8i1e52883_app2.pdf]

Online Intervention using Self-Compassionate Writing to Induce Positive Mood in Family Caregivers of Older Adults

Appendix 2

One-Way ANOVA scores with means and standard deviations for all conditions: Study 1 (*N* = 206)

Scale	*F*	*dfs*	*P*	Partial eta^2	Control *M (SD)*	Mind *M (SD)*	Kind *M (SD)*	CH *M (SD)*
Serenity	1.55	3,202	.20	.022	2.73(1.10)	2.51(0.93)	2.95(1.07)	2.78(1.17)
Guilt	3.40	3,202	.02[b]	.048	1.97(0.96)	2.33(1.02)	1.75(0.88)	1.97(0.91)
Sadness	2.78	3,202	.04[b]	.040	2.49(0.98)	2.68(1.22)	2.10(0.95)	2.51(1.06)
SSCS-S	1.13	3,198	.34	.017	3.12(0.73)	2.95(0.81)	3.24(0.87)	3.10(0.83)

Notes: CH – Common Humanity; Kind – Kindness; Mind – Mindfulness; *dfs* - degrees of freedom; SSCS-S – Self-Compassion Scale - Short Form; [b] Statistically significant *P* < .05.
