# Supplementary material for: Web-Based Intervention Using Self-Compassionate Writing to Induce Positive Mood in Family Caregivers of Older Adults: Quantitative Study
Source: JMIR Form Res. 2024 Nov 21;8:e52883. doi: 10.2196/52883 (PMC11621718; doi:10.2196/52883)
Supplement: Multimedia Appendix 3 [file formative_v8i1e52883_app3.pdf]

Online Intervention using Self-Compassionate Writing to Induce Positive Mood in Family Caregivers of Older Adults

Appendix 3

Correlations for scale composites: Study 2 (N = 224)

Scale	M (SD)	1	2	3	4	5	6	7	8	9
1. Serenity	2.39 (6.89)	-	-	-	-	-	-	-	-	-
2. Guilt	2.06 (1.03)	-.13	-	-	-	-	-	-	-	-
3. Sadness	2.38 (1.08)	-.15 [b]	.65[b]	-	-	-	-	-	-	-
4. Kindness	2.88 (0.98)	.09	-.46 [a]	-.43 [a]	-	-	-	-	-	-
5. Judgement	3.21 (0.99)	.05	-.59 [a]	-.55 [a]	.49 [a]	-	-	-	-	-
6. CH	3.25 (0.91)	-.01	-.13	-.12	.42 [a]	.09	-	-	-	-
7. Isolation	3.36 (1.11)	.01	-.52 [a]	-.70 [a]	.53 [a]	.66	.25	-	-	-
8. Mindful	3.17 (0.83)	.17 [b]	-.41 [a]	-.42 [a]	.70 [a]	.47 [a]	.51 [a]	.45 [a]	-	-
9. Over-Id	3.38 (0.89)	.10	-.51 [a]	-.46 [a]	.37	.67	.01	.61 [a]	.36 [a]	-
10. SSCS-L	3.21 (0.70)	.88	-.60 [a]	-.62 [a]	.80 [a]	.78 [a]	.51 [a]	.82 [a]	.78 [a]	.69 [a]

Notes: CH – Common Humanity; Judgement – Self-Judgement; Kindness – Self-Kindness; Mindful – Mindfulness; Over-Id – Over-Identification; SSCS-L – Self-Compassion Scale – Long Form.

[a] Statistically significant P < .001; [b] Statistically significant P < .05.
