# Supplementary material for: Web-Based Intervention Using Self-Compassionate Writing to Induce Positive Mood in Family Caregivers of Older Adults: Quantitative Study
Source: JMIR Form Res. 2024 Nov 21;8:e52883. doi: 10.2196/52883 (PMC11621718; doi:10.2196/52883)
Supplement: Multimedia Appendix 4 [file formative_v8i1e52883_app4.pdf]

Online Intervention using Self-Compassionate Writing to Induce Positive Mood in Family Caregivers of Older Adults

Appendix 4

One-Way ANOVA scores with means and standard deviations for all conditions: Study 2 (*N* = 224)

Scale	*F*	*P*	*Partial eta*^{2}	Control *M (SD)*	Mindful *M (SD)*	Kindness *M (SD)*	CH *M(SD)*
Serenity	1.15	.33	.015	2.80(1.16)	0.92(13.52)	2.94(1.09)	2.91(1.02)
Guilt	1.36	.26	.018	2.20(1.11)	2.18(0.98)	1.86(0.92)	1.20(1.09)
Sadness	0.87	.23	.003	2.43(1.02)	2.44(1.13)	2.36(0.99)	2.29(1.19)
Kindness	1.73	.16	.023	2.83(0.95)	2.66(1.02)	3.05(0.91)	2.98(1.02)
Judge	1.39	.25	.019	3.12(1.03)	3.05(0.95)	3.37(0.93)	3.32(1.03)
CH	0.20	.90	.003	3.31(0.81)	3.23(0.93)	3.19(0.98)	3.28(0.95)
Isolation	0.49	.69	.007	3.47(1.08)	3.24(1.16)	3.31(1.10)	3.42(1.10)
Mind	0.94	.42	.013	3.24(0.79)	3.06(0.83)	3.28(0.76)	3.09(0.94)
Over-Id	0.38	.77	.005	3.33(0.89)	3.34(0.88)	3.49(0.92)	3.38(0.91)
SSCS-L	0.73	.53	.010	3.23(0.71)	3.10(0.72)	3.28(0.62)	3.24(0.75)

Notes: CH – Common Humanity; Degrees of freedom – 3,220 on all measures; Judge – Self-Judgement; Kindness – Self-Kindness; *M(SD)* - Means and standard deviations by condition; Mindful – Mindfulness; Over-Id – Over-Identification; SSCS-L Self-Compassion Scale – Long Form.
