# Supplementary material for: Web-Based Intervention Using Self-Compassionate Writing to Induce Positive Mood in Family Caregivers of Older Adults: Quantitative Study
Source: JMIR Form Res. 2024 Nov 21;8:e52883. doi: 10.2196/52883 (PMC11621718; doi:10.2196/52883)
Supplement: Multimedia Appendix 5 [file formative_v8i1e52883_app5.pdf]

Online Intervention using Self-Compassionate Writing to Induce Positive Mood in Family Caregivers of Older Adults

Appendix 5

Correlations for Scale Composites Times 1 and 2: Study 3 (*N* = 222)

Scale: Time 1	M (SD)	1	2	3	4	5	6	7	8	9
1. Serenity	2.70 (1.06)	-	-	-	-	-	-	-	-	-
2. Guilt	2.15 (1.04)	-.51[a]	-	-	-	-	-	-	-	-
3. Sadness	2.57 (1.12)	-.58 [a]	.70 [a]	-	-	-	-	-	-	-
4. Kindness	2.63 (0.91)	.49 [a]	-.41 [a]	-.50 [a]	-	-	-	-	-	-
5. Judgement	3.02 (0.93)	.40 [a]	-.64 [a]	-.53 [a]	.55 [a]	-	-	-	-	-
6. CH	3.30 (0.86)	.28 [a]	-.33 [a]	-.35 [a]	.44 [a]	.41 [a]	-	-	-	-
7. Isolation	3.09 (1.04)	.50 [a]	-.54 [a]	-.71 [a]	.52 [a]	.62 [a]	.40 [a]	-	-	-
8. Mindfulness	3.10 (0.86)	.47 [a]	-.52 [a]	-.50 [a]	.62 [a]	.57 [a]	.58 [a]	.52 [a]	-	-
9. Over-Id	3.27 (0.88)	.37 [a]	-.46 [a]	-.42 [a]	.36 [a]	.63 [a]	.27 [a]	.50 [a]	.57 [a]	-
10. SSCS-L	3.07 (0.70)	.55 [a]	-.63 [a]	-.66 [a]	.76 [a]	.83 [a]	.66 [a]	.79 [a]	.83 [a]	.72 [a]
Scale: Time 2	M (SD)	1	2	3	4	5	6	7	8	9
1. Serenity	2.70 (0.96)	-	-	-	-	-	-	-	-	-
2. Guilt	1.94 (1.03)	-.51 [a]	-	-	-	-	-	-	-	-
3. Sadness	2.36 (1.05)	-.61 [a]	.71 [a]	-	-	-	-	-	-	-
4. Kindness	2.99 (0.91)	.45 [a]	-.42 [a]	-.51 [a]	-	-	-	-	-	-
5. Judgement	3.32 (0.97)	.42 [a]	-.62 [a]	-.54 [a]	.50 [a]	-	-	-	-	-
6. CH	3.66 (0.84)	.26 [a]	-.23 [a]	-.30 [a]	.44 [a]	.28 [a]	-	-	-	-
7. Isolation	3.37 (1.12)	.57 [a]	-.55 [a]	-.79 [a]	.51 [a]	.62 [a]	.37 [a]	-	-	-
8. Mindfulness	3.40 (0.80)	.48 [a]	-.47 [a]	-.50 [a]	.66 [a]	.59 [a]	.55 [a]	.48 [a]	-	-
9. Over-Id	3.53 (0.92)	.14 [a]	-.47 [a]	-.46 [a]	.36 [a]	.68 [a]	.14 [b]	.48 [a]	.48 [a]	-
10. SSCS-L	3.38 (0.70)	.58 [a]	-.62 [a]	-.71 [a]	.76 [a]	.83 [a]	.60 [a]	.80 [a]	.82 [a]	.70 [a]

Notes: CH - Common Humanity; Judgement – Self-Judgement; Kindness – Self-Kindness; Over-Id - Over-Identification; SSCS-L – Self-Compassion Scale – Long Form.

[a] Statistically significant $P < .001$; [b] Statistically significant $P < .05$.
