# Supplementary material for: Web-Based Intervention Using Self-Compassionate Writing to Induce Positive Mood in Family Caregivers of Older Adults: Quantitative Study
Source: JMIR Form Res. 2024 Nov 21;8:e52883. doi: 10.2196/52883 (PMC11621718; doi:10.2196/52883)
Supplement: Multimedia Appendix 6 [file formative_v8i1e52883_app6.pdf]

Online Intervention using Self-Compassionate Writing to Induce Positive Mood in Family Caregivers of Older Adults

Appendix 6

Repeated measures ANOVA scores for Condition x Time effects with means and standard deviations by condition: Study 3 (*N* = 222)

Scale	*F*	*dfs*	*P*	*Eta* S^2		
Serenity	4.55	2,218	.01 [b]	.040		
Guilt	9.85	2,219	< .001	.083	-	-
Sadness	11.48	2,219	< .001	.095	-	-
Kind	6.10	2,219	.003 [b]	.053	-	-
Judg	0.53	2,219	.59	.005	-	-
CH	4.59	2,219	< .001	.040	-	-
Isol	1.02	2,219	.36	.009	-	-
Mind	0.80	2,219	.45	.007	-	-
Over-Id	1.00	2,219	.37	.009	-	-
SSCS-L	2.12	2,219	.12	.019	-	-

			Condition			
	C 1	C 2	SC 1	SC 2	WM 1	WM 2
Scale	*M (SD)*	*M (SD)*	*M (SD)*	*M (SD)*	*M (SD)*	*M (SD)*
Serenity	2.76(1.06)	2.55(0.91)	2.70(1.03)	2.76(0.93)	2.63(1.09)	2.80(1.02)
Guilt	2.13(0.96)	2.22(1.09)	2.09(0.97)	1.78(0.87)	2.24(1.17)	1.83(1.07)
Sadness	2.46(1.13)	2.56(1.15)	2.59(1.05)	2.33(.094)	2.65(1.17)	2.18(1.04)
Kind	2.62(0.91)	2.72(0.93)	2.66(0.97)	3.16(0.92)	2.62(0.86)	3.09(0.84)
Judg	2.93(0.88)	3.30(1.03)	3.15(0.99)	3.44(0.96)	3.00(0.92)	3.23(0.92)
CH	3.26(0.89)	3.41(0.92)	3.30(0.84)	3.71(0.79)	3.34(0.85)	3.86(0.76)
Isol	3.03(1.06)	3.21(1.19)	3.15(1.06)	3.49(1.05)	3.11(1.02)	3.41(1.11)
Mind	2.96(0.89)	3.26(0.88)	3.21(0.83)	3.44(0.79)	3.12(0.85)	3.49(0.73)
Over-Id	3.26(0.88)	3.42(0.96)	3.30(0.93)	3.63(0.93)	3.26(0.86)	3.53(0.87)
SSCS-L	3.01(0.73)	3.22(0.77)	3.13(0.72)	3.48(0.68)	3.07(0.65)	3.43(0.62)

Notes: CH – Common Humanity; *dfs* – degrees of freedom; Isol – Isolation; Judg– Self-Judgement; Kind – Self-Kindness; Mind – Mindfulness; Over-Id– Over-identification; SSCS-L – Self-Compassion Scale – Long Form; Condition – C 1 and C 2 – Control time 1 and 2; SC 1 and SC 2 – Self-Compassion time 1 and 2; WM 1 and WM 2 – Self-Compassion Without Mindfulness time 1 and 2; [a] Statistically significant *P* < .001; [b] Statistically significant P < .05.
