# Supplementary material for: Web-Based Intervention Using Self-Compassionate Writing to Induce Positive Mood in Family Caregivers of Older Adults: Quantitative Study
Source: JMIR Form Res. 2024 Nov 21;8:e52883. doi: 10.2196/52883 (PMC11621718; doi:10.2196/52883)
Supplement: Multimedia Appendix 7 [file formative_v8i1e52883_app7.pdf]

Online Intervention using Self-Compassionate Writing to Induce Positive Mood in Family Caregivers of Older Adults

Appendix 7

Study 3 Simple Slope Change (from Time 1 pre-manipulation to Time 2 post-manipulation) as a Function of each Condition (Control, Self-Compassion, and Self-Compassion Without Mindfulness), Separately (*N* = 222)

	Control			
	F	*dfs*	*P*	*Partial eta²*
Serenity	6.83	1,74	.01 [b]	.084
Guilt	0.81	1,74	.37	.001
Sadness	1.15	1,74	.29	.015
Kindness	1.38	1,74	.24	.018
Judgement	14.51	1,74	< .001	.164
CH	4.87	1,74	.03 [b]	.062
Isolation	4.34	1,74	.04 [b]	.055
Mindfulness	18.96	1,74	< .001	.204
Over-Id	3.28	1,74	.07	.042
SSCS-L	13.92	1,74	< .001	.158
	Self-Compassion			
	F	*dfs*	*P*	*Partial eta²*
Serenity	0.51	1,71	.51	.006
Guilt	12.56	1,72	< .001	.149
Sadness	11.78	1,72	< .001	.141
Kindness	23.86	1,72	< .001	.249
Judgement	10.65	1,72	.002 [b]	.129
CH	19.80	1,72	< .001	.216
Isolation	14.84	1,72	< .001	.171
Mindfulness	7.09	1,72	.01 [b]	.090
Over-Id	11.62	1,72	< .001	.139
SSCS-L	31.62	1,72	< .001	.305
	Self-Compassion Without Mindfulness			
	F	*dfs*	*P*	*Partial eta²*
Serenity	2.69	1,73	.11	.035
Guilt	37.45	1,73	< .001	.339
Sadness	30.02	1,73	< .001	.291
Kindness	28.78	1,73	< .001	.283
Judgement	4.25	1,73	.04 [b]	.055
CH	27.85	1,73	< .001	.276
Isolation	13.52	1,73	< .001	.156
Mindfulness	20.53	1,73	< .001	.219
Over-Id	8.72	1,73	.004 [b]	.107
SSCS-L	40.14	1,73	< .001	.355

Notes: CH – Common Humanity; *dfs* – degrees of freedom; Judgment – Self-Judgement; Kindness – Self-Kindness; Over-Id– Over-identification; SSCS-L – Self-Compassion Scale – Long Form; [b] Statistically significant $P < .05$.
